# A review of algorithmic approaches for cell culture media optimization

**DOI:** 10.3389/fbioe.2023.1195294

**Published:** 2023-05-11

**Authors:** Tianxun Zhou, Rinta Reji, Ryanjit Singh Kairon, Keng Hwee Chiam

**Affiliations:** ^1^ Bioinformatics Institute, Cellular Image Informatics Division, A*STAR, Singapore, Singapore; ^2^ School of Biological Sciences, Nanyang Technological University, Singapore, Singapore

**Keywords:** culture media optimization, media optimization algorithm, fermentation optimization, surrogate model, metaheuistics, design of experiements, benchmark comparison

## Abstract

Cell culture media composition and culture conditions play a crucial role in product yield, quality and cost of production. Culture media optimization is the technique of improving media composition and culture conditions to achieve desired product outcomes. To achieve this, there have been many algorithmic methods proposed and used for culture media optimization in the literature. To help readers evaluate and decide on a method that best suits their specific application, we carried out a systematic review of the different methods from an algorithmic perspective that classifies, explains and compares the available methods. We also examine the trends and new developments in the area. This review provides recommendations to researchers regarding the suitable media optimization algorithm for their applications and we hope to also promote the development of new cell culture media optimization methods that are better suited to existing and upcoming challenges in this biotechnology field, which will be essential for more efficient production of various cell culture products.

## 1 Introduction

Cell culture is widely used in biotechnology to manufacture various useful products for applications such as pharmaceuticals, food, biofuel, and industrial products. Cell culture production systems in industry and research span different kingdoms of life from free living microbes such as bacteria, archaea, and fungi, to cell lines derived from multicellular organisms including insect and mammalian species. Products include pharmaceuticals such as antibiotics and monoclonal antibodies; food products such as rennet, single-cell protein, and cultivated meat; biofuel from lipid-producing algae; industrial products such as cleaning enzymes and organic acids.

In cell culture, culture media is a crucial input that provides energy and materials required by cells to grow, proliferate and produce the products of interest. The components that make up culture media and their concentrations within the media affect important aspects of cell growth, productivity and product quality, and are instrumental to the success of the cell culture application (Yao and Asayama, 2017; Ritacco et al., 2018). In most laboratory applications, a universal standardized media is used but to achieve cost-efficient upscaling to industrial production, there is a need to identify a combination of concentrations for media components that optimize for the desired property, such as biomass or specific biomolecule yield in the cell culture system. There have been many methods using optimization algorithms developed for culture media optimization for both microbial systems and animal cell culture. Despite this, there is a lack of systematic review and comparison of these different methods from an algorithmic perspective that provides researchers with a comprehensive overview of the available algorithms and provides perspectives on the applicability of these methods in new contexts.

A few other review articles are available on culture media optimization. The review by Singh et al. (2017) focuses on fermentation media for microbial systems. While it lists some of the techniques used in literature, it does not include several types of algorithms that were typically used in media optimization, and also lacked a benchmark comparison between the methods. Similarly, the reviews by van der Valk et al. (2010) Ritacco et al. (2018) and Galbraith et al. (2018) does not go into depth on the available optimization algorithms. The unique requirements of the culture media optimization problem, such as the need for a low number of iterations due to experimental resource constraints, and noisy results are also unaddressed by reviews of optimization algorithms for black-box functions that evaluate algorithms in a general context.

The purpose and unique contribution of this review is to provide readers with a comprehensive overview of the main types of algorithms applicable to culture media optimization. We synthesized existing works and provided a generalized framework for understanding and designing culture media optimization experiments. We have examined and summarized the strategies adopted by previous works, classify and explain them using this framework and examine algorithmic features that were designed and chosen during past efforts to address specific challenges of this problem. We have also provided recommendations on the type of algorithm to use based on benchmark comparisons and identify gaps for future research.

## 2 Materials and methods

### 2.1 Scope of review

The cell culture media optimization works covered by this review apply to both microbial and animal cell culture for a variety of outcomes including maximizing biomolecule yield, biomass production and cell proliferation. The methods reviewed cover algorithmic approaches for optimization that have been used in literature for optimizing culture media conditions.

Simple methods such as one-factor-at-a-time (OFAT) and factor screening through statistical design of experiment (DOE) are not covered in this review. These methods are relatively simple and standard and are well discussed in other works. These methods are also generally recognized to be insufficient for more complex media formulations that contain more components (more than 10) and concentration levels due to combinatorial explosion, and potential complex interaction effects that exist between components.

Bioinformatics methods such as metabolic network models and expression analysis are also excluded from the review. These methods require specific and customized analysis and modeling for the cell line and process involved.

### 2.2 Methodology of review

We carried out a systematic literature search for cell culture media on NCBI PubMed and Google Scholar with the query:

• ((“Cell culture” OR “Culture” OR “fermentation”) AND (“media” OR “medium”) AND “optimization”) AND (algorithm[Text Word])

We also conducted a literature search for specific algorithms that we are aware have been or may be applied to the media optimization problem by replacing the generic term “algorithm” in the search with the following keywords:

• iterative
• direct search
• simplex
• metaheuristics
• differential evolution
• evolutionary strategies
• swarm
• simulated annealing
• surrogate model
• regression
• kriging
• bayesian
• gaussian process
• support vector
• decision tree
• random forest
• ensemble
• neural network
• deep neural network
• deep learning
• machine learning
• artificial intelligence

### 2.3 Method for simulation experiment for benchmarking

Most available works in culture media optimization generally propose a single optimization algorithm for a particular cell culture experiment. It is difficult to draw conclusions on the effectiveness of each of these algorithms vis-a-vis other algorithms. This presents an obstacle for other researchers seeking to make informed choices on which algorithms to utilize in the face of experimental resource constraints.

Few of the papers reviewed provided code implementations of the algorithms. We default to open-source implementations of algorithms written in Python if available. Otherwise, we modify open-source code or write our own implementations to recreate the algorithms. The codes for the simulation experiment for this study can be found at https://github.com/zhoutianxun/Review-of-culture-media-optimization-methods.

To provide a benchmark comparison of the algorithms, we propose to compare them on a large set of test functions with different characteristics. The test functions used come from the Black-Box Optimization Benchmarking (BBOB) test suite (Hansen et al., 2021). Details about the test functions are given in Supplementary Table S1.

For our main experiments, we used the 5, 20 and 40-dimension versions of the test functions to represent typical media optimization experiments. For each individual experiment, 10 runs were performed with a population size of 50. The methods were only run to a maximum of 10 iterations to model the constraints of typical cell culture optimization experiments.

To generate noisy functions, we simulated additive Gaussian noise by sampling from a Gaussian distribution and adding that to the true function value. Additive noise was used to better simulate the measurement uncertainty from assays used to quantify the yield of interest in a cell culture experiment.

## 3 Classification of algorithmic approaches

### 3.1 Basic terminology

There are many terminologies used in culture media optimization literature that may be confusing to newcomers in the field, originating from different sources from the fields of experiment design, mathematical optimization, statistics, evolutionary computation, machine learning and others.

Here we define some of the basic terminologies that would be encountered in culture media optimization literature, with the most common names in bold:

Common terminology.

• The adjustable components in the culture media
  – **Component, Factor, Parameter, Variable**, Input, Dimension, Features
• The value of the factor. Levels are used specifically for discretized factor value
  – **Concentration, Level, Value**
• A list of culture media candidates, each with varying values for the factors
  – **Design, Candidates**, **Population**, Experiments, Runs, Formulation
• The target value to be optimized for, obtained through experiment
  – **Response, Objective value,** Fitness, Output, Read-out
• The combined set of culture media candidates and their corresponding response obtained through experiment
  – **Data(point), Results,** Training Set
• In an iterative optimization workflow, each batch of experiment
  – **Iteration, Generation, Round,** Batch

### 3.2 A generalization of media optimization methods

**FIGURE 1:**
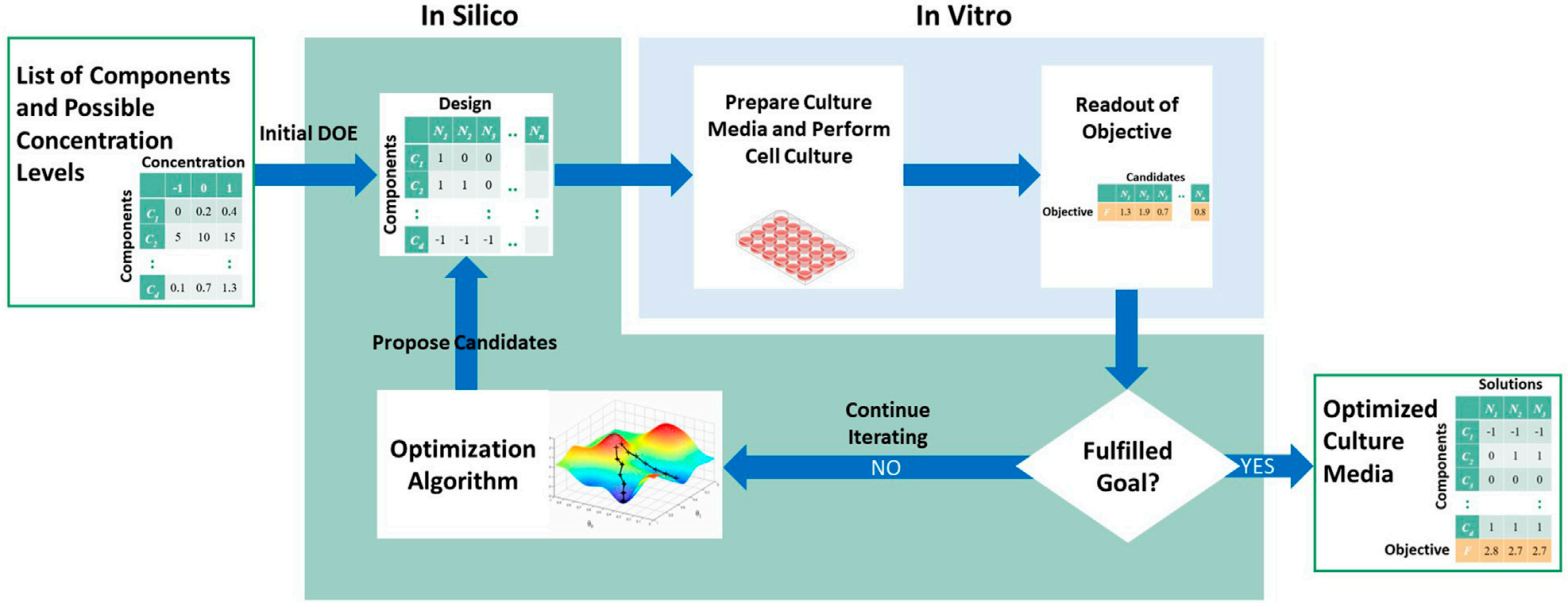
Generalization of media optimization methods.

The methods for media optimization can be generalized as an iterative computational-experimental workflow (Figure 1). In this general workflow, a list of components and their range of values are defined prior to the optimization process. For each iteration, the optimization algorithm is used to propose a list of candidates, also known as the experiment design. Note that in the first iteration, the initial list of candidates, known as the initial design of experiment, is proposed not by the optimization algorithm but rather using standard designs or random sampling from the input space. Next, the culture media corresponding to these candidates are prepared and cells are cultured in each candidate media. A measure of the objective of interest, such as protein yield, is quantified experimentally. These values are then fed back to the optimization algorithm to propose the next iteration of candidates. Alternatively, the workflow terminates if no more improvement is observed, or if experimental budget is reached.

Thus, based on this general workflow, we can divide a media optimization method into several parts, and classify existing methods based on these parts:

1. Problem definition, i.e., optimization objective and media component space
2. Initial design of experiment
3. Optimization algorithm

### 3.3 Problem definition

#### 3.3.1 Objective type

Optimization can be classified based on the objective as single-objective or multi-objective. Single-objective is the most common type, especially if the value of the product greatly exceeds the cost of the culture media, in which case optimizing for yield alone is sufficient. Multi-objective optimization is used when multiple outcomes of interests are considered. For example, maximizing the yield of a useful product while minimizing the production of a toxic metabolite, or maximizing yield while minimizing the cost of culture media.

For multi-objective optimization, a range of possible optimal solutions exists known as the Pareto front. A Pareto optimal solution is a point where it is not possible to improve one objective without worsening another.

When trying to optimize culture media with multiple objectives in mind, it can either be tackled as a multi-objective problem or converted to a single-objective problem by modifying the objective function. For example, to balance the goal of increased cell proliferation, low cost and ease of use, Cosenza et al. (2021) used a single objective function that normalizes the measure of cell proliferation by the volume of fetal bovine serum (FBS) which is the costly component used in the media.

#### 3.3.2 Factor value type

The values of the input factors of the culture media can be classified as continuous values or discrete levels. Discrete levels simplify the problem by reducing possible combinations to a finite set. However, continuous values allow for a finer-grained optimization of the factors.

In some cases, by increasing the number of levels, it is possible to approximate a continuous value with discrete levels such as in Havel et al. (2006) where 7 binary bits resulting in 128 levels were used to represent the values for a discrete genetic algorithm. Vice-versa, the opposite can be achieved by rounding off continuous values to fixed discrete levels as seen in Kim & Audet (2019). This can be useful when adapting algorithms that were designed originally for discrete or continuous problems to the requirement of the problem.

In the works reviewed, a few (2–3) discrete levels are commonly adopted during the initial design of experiment. In subsequent iterations, continuous values are often employed for more precise media formulations.

#### 3.3.3 Choice of factors

There are many important factors that affect cell proliferation and metabolite production, and these factors change according to the desired outcome of the optimization. However, not all media components contribute significantly to the desired outcome and changing concentrations of these components are inconsequential. Thus, it is important to omit such factors from the optimization strategy to prevent the redundant and excessive use of laboratory resources. One strategy used by many researchers is to conduct a screening of factors using DOE methods such as Plackett-Burman design or Definitive Screening design. This serves to identify the most important factors that affect the response, thus reducing the input space to a more manageable dimension. The use of statistical DOE for factor screening is well established and we refer interested readers to Antony (2014). Considerations on the number of factors given experimental budget constraint are further discussed in Section 7.

### 3.4 Initial design of experiment

An experiment design refers to a list of candidates that have varying values for each of the factors.

In the first iteration, an initial design of experiment (DOE) is conducted without the need for the optimization algorithm as a starting point for the optimization problem, assuming that no data is available yet. Designs can be classified into two types, statistical DOE and random designs. It is also possible to have a mixture of both by supplementing statistical designs with some random designs.

### 3.5 Optimization algorithm

**FIGURE 2:**
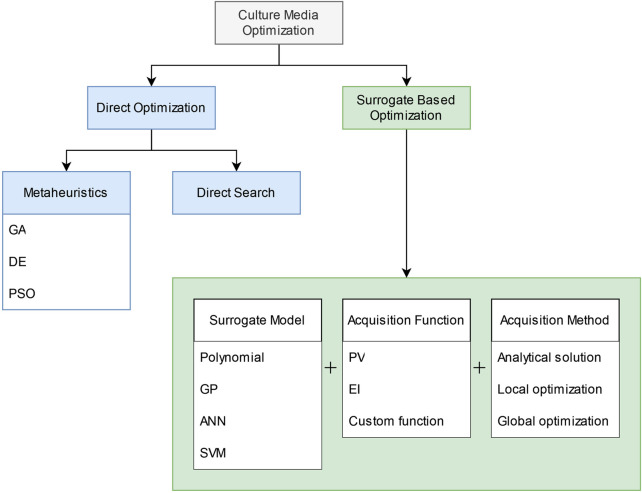
Classification of optimization algorithms used in media optimization literature.

The types of algorithms used in cell culture media optimization works can be broadly classified into two classes: direct optimization, and surrogate-based optimization (Figure 2).

We define direct optimization algorithms as methods that optimize by evaluating the objective function directly. Direct search and metaheuristics algorithms are two types of methods that fall under our definition of direct optimization.

Direct search methods perform a sequential examination of trial solutions, involving a comparison of each trial solution with the best solution obtained up to that time (Audet and Hare, 2017). A strategy is used to determine what the next trial solution will be. Direct search methods are deterministic in nature. Commonly used direct search methods include Nelder-Mead downhill simplex, pattern search, and mesh adaptive direct search. None of the papers reviewed used direct search methods to directly optimize culture media. However, direct search methods have been used as acquisition methods in surrogate-based optimization, which will be discussed in Section 6.3.

Metaheuristics optimization algorithms are designed with heuristics or strategies for generating candidates to find good solutions to an optimization problem (Audet and Hare, 2017). Metaheuristics algorithms are often inspired by naturally occurring optimizing phenomena, such as the optimization of fitness through evolution by natural selection by life, the efficient foraging of food by animal swarms or the reordering of atoms into lower energy states through the process of annealing. In metaheuristics optimization, the objective function values of candidates are used as information to inform the generation of new candidates by applying heuristic rules. They also contain elements of stochasticity that helps to avoid being trapped in local optima. This characteristic of stochasticity is a key difference compared to direct search methods. Commonly used metaheuristics optimization algorithms include genetic algorithm, simulated annealing and particle swarm algorithm.

Surrogate-based optimization (SBO) is the other major class of optimization algorithms. SBOs were initially conceptualized as a way of optimizing computationally expensive simulation experiments but have since been used to optimize black-box functions in general (Koziel et al., 2011). A surrogate model is used to estimate the true objective function by training on a relatively small set of samples obtained from the true function. Because the surrogate model is much cheaper to evaluate than the true function, it can be queried more extensively and used to suggest candidates for further testing. The surrogate model is trained on the initial DOE inputs and the experimentally obtained outputs. Using the surrogate model as an estimator of the true objective function, new candidate solutions are proposed based on a certain strategy. The new candidate is then evaluated experimentally, and the new data is used to update the surrogate model. The process is repeated until the result is satisfactory or if the experiment budget is exceeded. Commonly used surrogate models include quadratic response surface method (RSM), Kriging (also known as Gaussian process regression), and neural networks.

Other than the two classes described above, within optimization literature hybrid methods are also used.Hybrid methods combine aspects of both metaheuristics and surrogate based optimization (Stork et al., 2022). This may be achieved in several ways. For example, different algorithms can be run simultaneously and proposed candidates combined; or during the optimization iteration, one control the outer loop, while another method is used subsequently in the inner loopbe nested in another algorithm. In the culture media optimization literature surveyed, we have not come across studies that used hybrid methods. Some studies perform sets of experiment using metaheuristics and SBO in parallel to compare their performance, but the candidates are not combined between the two sets.

## 4 Initial design of experiment

### 4.1 Statistical design of experiment (DOE) designs

Statistical Design of Experiments (DOE) is a method used to systematically investigate the relationship between variables in a controlled experimental setting. Several types of DOE designs are available and can be used for the initial design (Jankovic et al., 2021).

Full factorial design: This design includes all possible combinations of the levels of each variable. While this method is the most comprehensive, it is unfeasible for media with many components, as the total number of experiments would be x^k^ where x is the number of levels and k is the number of components.

Fractional factorial design: This design includes a fraction of all the possible combinations of levels of each variable. The use of this design results in more efficient use of resources but result in confounding with interaction terms.

Taguchi design: This is a design that is an improvement to full and fractional factorial designs. It involves using orthogonal arrays to arrange the factors affecting the experiment and determine the levels at which they should be set. In contrast to traditional DOE, Taguchi treats noise as a focus of analysis (Hernadewita et al., 2019).

Central composite design (CCD): This design method starts with an embedded factorial or fractional factorial design with center points and adds “star” points to estimate curvature.

Box-Behnken Design (BBD): This design, unlike central composite design, is an independent quadratic design that does not contain an embedded factorial or fractional factorial design. The component combinations are the midpoints of edges of the process space and the center point. Compared to central composite design, the Box-Behnken design has limited capability for orthogonal blocking.

### 4.2 Random designs

When a large number of factors are present, statistical DOE designs are often infeasible to be carried out as the number of experiments required grows exponentially. An alternative to DOE designs is random designs.

A simple random sampling method that samples from a uniformly random distribution for each factor is non-ideal as it tends to result in uneven distances between samples in the input space. This means some parts of the input space are inadequately sampled and reduces the effectiveness of subsequent optimization. In comparison, space filling designs are able to evenly cover the input space with samples.

The most commonly used space filling design is the Latin hypercube design (LHS). Latin hypercube samples are generated such that each hyperplane of the input space contains only one sample. LHS is used by a few works (Cosenza et al., 2022; Yoshida et al., 2022) as the initial DOE.

## 5 Metaheuristics optimization

Following the taxonomy proposed by Stork et al. (2022), metaheuristics optimization can be broadly classified as population type, hill climbing type and trajectory type. For iterative experiments in cell culture experiment settings, the use of automated liquid handlers to prepare solutions allows multiple candidates to be tested in each iteration. Population-type algorithms are most suitable to leverage this capability and provide faster optimization.

Popular population-type metaheuristics include evolution-inspired algorithms such as genetic algorithm, evolutionary strategies, and differential evolution, and swarm-inspired algorithms such as particle swarm algorithm and ant-colony algorithm. We will introduce the algorithms that have been applied for cell culture optimization. For more in-depth details on metaheuristics optimization, we refer interested readers to Du and Swamy (2016).

### 5.1 Genetic algorithm

Genetic algorithm (GA) is a metaheuristics optimization inspired by the process of evolution through natural selection. It generates new candidates through biologically inspired operations of selection, crossover and mutation.

In the classic GA, candidates are represented as a “chromosome”, where the value of each factor, in discrete levels, is coded as a bit string. In each iteration, three steps take place to generate the subsequent population of candidates (Mirjalili, 2019).

1. Selection, which selects some candidates of the previous generation as “parents”. By the analogy of natural selection where fitter candidates survive more to give rise to offspring, preference is given to candidates that score highly on the objective function.
2. Crossover, where “parents” are combined to produce new “offspring” candidates. Random sites are chosen on the “chromosome” to be retained on the offspring candidate
3. Mutation, where random changes occur to the offspring candidate to create more diversity and to prevent premature convergence to local optima.

Many variants of GA exist, with different definitions of the three operations. The classic GA is designed for discrete problems as each input is represented as a string. However, GA has also been adopted for continuous problems, by adopting a continuous “chromosome” representation, and modifying the crossover and mutation operations.

GA is a popular method in culture media optimization. It has been often used for problems that have many factors. GA is also one of the most popular methods used as the acquisition method in surrogate-based optimization.

### 5.2 Differential evolution

Differential evolution (DE) is another evolution-inspired metaheuristics optimization that is popular, especially for continuous problems. DE also generates new candidates through the biologically inspired operation of mutation and crossover like GA but instead of strings, the inputs are represented as real-valued vectors, implemented as floats.

In classic DE, in each iteration, three steps take place to generate the new population of candidates (Price, 2013).

1. Mutation is achieved by selecting a random target vector and adding it to a scaled difference of two other randomly selected solution vectors, to generate a trial vector.
2. Crossover is achieved by discrete recombination of the parent vector and trial vector with a given crossover probability P_CR_, to obtain the offspring vector.
3. After evaluating the offspring vectors in the objective function, the fitter one between the offspring and parent vector is retained for the next iteration.

Advanced variants of DE such as L-SHADE have been proposed in black-box optimization literature. DE optimizes continuous problems by default but can be adapted to discrete problems easily by rounding off candidates to their nearest discrete level. Variants of DE have been applied to optimize media components in some studies.

### 5.3 Particle swarm optimization

Particle swarm optimization (PSO) is a metaheuristics optimization inspired by mimicking animal flocking behavior. Each particle’s movement is influenced by both its personal best-known position and the best-known positions of the swarm as a whole, in the expectation that the swarm as a whole moves towards the global optimum (Kennedy and Eberhart, 1995). The algorithm starts with a population, also known as a swarm, of candidate solutions.

• Each particle (candidate solution) moves around the search space.
• The current location *x*_*id*_ and velocity *v*_*id*_ and personal best *pbest*_*i*_ of any particle *i* and the global best *gbest* of the entire population are used to compute how the particles should move next in the *d*-dimensional hyperspace.

This process is repeated until a satisfactory optimum is reached. A few papers have used this approach(Cockshott and Hartman, 2001; Huang et al., 2007; Garlapati et al., 2010; Khaouane et al., 2012).

## 6 Surrogate-based optimization

Surrogate-based optimization (SBO) can be viewed as comprising three separate components: the surrogate model, the acquisition function, and the acquisition method (Forrester and Keane, 2009). The surrogate model refers to the model that is used to approximate the actual function of interest by fitting experimental data. Before the first iteration, the model is fitted with the data collected from the initial DOE. The acquisition function refers to the function or criterion that is used to decide which candidates to propose and evaluate in the next round of experiments. The acquisition method refers to how the candidates are found by optimizing the acquisition function. (Note: The acquisition of new points, i.e., both the choice of acquisition function and acquisition method may also be referred to as infill strategy or infill criteria in literature (Zhou et al., 2020). It is also closely related to the concept of active learning in machine learning, which seeks to improve the model’s accuracy with less training data by systematically acquiring training sample (Ren et al., 2021).

To illustrate with an example, a common SBO workflow used in media optimization is the ANN-GA method. In this method, the surrogate model used is an artificial neural network (ANN), that predicts the response given a valid media design as input. The ANN is trained with actual response values collected from experiments performed with the initial DOE candidates. As we assume that the ANN is an accurate predictor of the response, and we would like to maximize the response, we would then computationally find the input that maximizes the predicted response of the ANN. The acquisition function in this case would be the predicted value (PV) because we acquire new candidates based on the predicted value output by the surrogate model. These candidates are found by running GA on the ANN as the objective function. GA in this case would be the acquisition method. The acquired candidates that maximize the response of ANN are then evaluated experimentally and added to training data to update the ANN model in the next iteration.

We will introduce the common types of surrogate models, acquisition functions and acquisition methods (if they have not been covered in Section 5), that have been applied for cell culture optimization. We also refer interested readers to (Forrester and Keane, 2009, Jiang et al., 2020) for more in-depth resources on surrogate optimization.

### 6.1 Surrogate models

#### 6.1.1 Polynomial response surfaces

Response surface methodology (RSM) is a type of surrogate-based optimization method that was first introduced in 1951 and has found wide applications in optimizing industrial processes (Khuri and Mukhopadhyay, 2010). The typical RSM approach is to first determine significant factors with a linear model, known as factor screening. After which, only the significant factors are kept for optimization. A linear model is again used to find the suitable range of input where the optimal is likely to lie in, by going along the path of steepest ascent or descent. After which, a DOE is conducted typically with CCD or BBD, and the data is used to fit a higher-order polynomial model. The polynomial model, which is essentially a surrogate model, is then used for optimization.

The most common response surface is the second-order polynomial, based on the assumption that the landscape of the objective function can be approximated with a second-order Taylor expansion. The standard form of the model is given as

$$y=\beta_{0}+\sum_{j=1}^{m}\beta_{j}x_{j}+\sum_{j=1}^{m}\beta_{jj}x_{j}^{2}+\sum \sum_{j<k}^{m}\beta_{jk}x_{j}x_{k}$$

Polynomial response surfaces are easy to fit and computationally inexpensive. However, as they are relatively simple, empirically they are often less accurate than other types of surrogate models. When there are many factors involved, polynomial response surfaces are often inadequate in providing accurate predictions. In media optimization literature, polynomial response surfaces are mostly used for problems involving 5 factors or less.

Analytical solution to find the optima of the polynomial response surface through canonical and ridge analysis is one way to find the maxima/minima of the surrogate model, although other methods such as genetic algorithms are used as well, sometimes with better results.

Typical usage of RSM for optimization follows a one-step optimization process where the model is fitted with the DOE results and one set of candidates is proposed based on the model which is subsequently tested in the real-world experiment before the optimization process terminates. However, iterative refinement of the polynomial response surface is also possible. Although not in any of the reviewed culture media optimization literature that uses RSM, the iterative workflow has been used in other applications (Goswami et al., 2016).

#### 6.1.2 Gaussian process models

Gaussian process (GP) model, also known as Kriging model, is one of the most widely used surrogate models (Forrester and Keane, 2009), especially in engineering applications. GP-based surrogate model optimization is often known as Bayesian optimization (Snoek et al., 2012) in the machine learning literature used for optimizing model hyperparameters.

GPs are non-parametric probabilistic regression models that model a distribution over functions and are hence able to give confidence for predictions. This is a useful property that is used for acquisition functions. It first defines a prior over functions, which can be converted into a posterior distribution over functions once data is observed. The main idea of GPs is the assumption that subsets of the function’s values have a joint Gaussian distribution. This means that given a set of inputs, the corresponding outputs will be distributed according to a multivariate Gaussian distribution. The covariance of the joint distribution is computed with a kernel function, which can be thought of as a similarity measure between the inputs. When observations, i.e., training data are provided, we can condition upon these observations to update the prior and compute the posterior distribution. The prediction for input with an unknown function value is done by marginalizing the posterior distribution on that input and extracting the mean value. The variance will be the confidence of the prediction.

GPs can be computationally expensive for large datasets. However, compared to the much more expensive cell culture experiments, the computational cost of GPs does not pose a practical challenge.

#### 6.1.3 Artificial neural networks

Artificial neural networks (ANN) are an increasingly popular machine learning model that have been used in a wide variety of tasks. ANNs are universal function approximators that can be used to model arbitrary functions and this contributes to their success.

**FIGURE 3:**
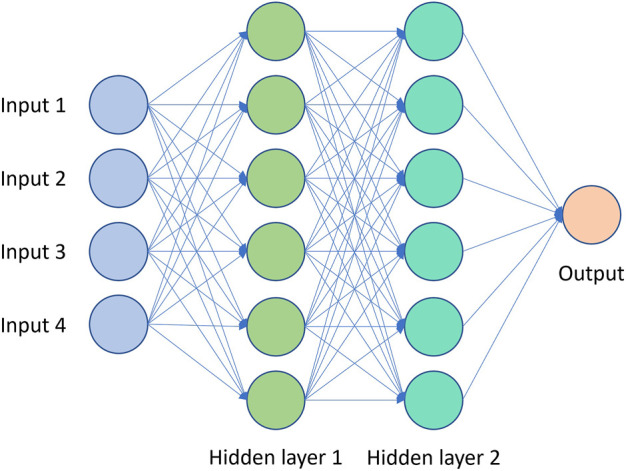
Diagram of typical Multi-Layer Perceptron.

ANNs are made up of neurons arranged in layers and connected together by weighted edges (Krogh, 2008). A simple feedforward network where each neuron in a layer is connected to all neurons in the previous layer, is known as a multi-layer perceptron (MLP) or a fully-connected neural network (FCNN) (Figure 3). The input, represented as a vector, is defined as the input layer, where each neuron takes on the value of one dimension of the input vector. The values of the neurons of each layer are computed by multiplying the value of the neurons in the previous layer by the weights of the edges connecting them to neurons of the current layer and summing them up. A non-linear function known as the activation function is applied to the value of each neuron which introduces non-linearity. For regression problems, no activation is applied to the final layer which outputs an unbounded continuous value.

Common activation functions include rectified linear unit (ReLU), sigmoid function, and hyperbolic tangent function. Radial basis functions can also be used as activation functions, and such neural networks, normally with single hidden layer are called radial basis neural networks (RBFNN) (Ghosh and Nag, 2001). A variation of RBFNN is the Generalized regression neural network (GRNN).

ANNs, specifically MLPs generally with 1 - 2 hidden layers are common surrogate models used in culture media optimization literature. There have been many works that compare ANNs and second-order polynomial response surfaces on the closeness of fit and RMSE of prediction on response to cell culture media. The general conclusion from those works is that ANNs are more accurate surrogate models and provide better optimization performance. RBFNNs are also popularly used as surrogate models in culture media optimization.

Deeper ANNs with many hidden layers, also known as deep learning models, are less common in media optimization. Two studies have used ANNs with 4 hidden layers (Tachibana et al., 2021; Yoshida et al., 2022) as surrogate models to predict the expression of GFP in *E. coli*. Having more layers increases the ability of the network to fit data, however it should be noted that such overparameterized models generally require a high number of training datapoints to prevent overfitting and poor generalization. Hence, having a validation set to test the performance of the model after training can be useful to determine if overfitting occurs, and to decide if a deep network is necessary by comparing the prediction performance against other types of surrogate models.

#### 6.1.4 Support vector machines

Support vector machines (SVM) are a type of machine learning model that learns a hyperplane that has the greatest number of training points falling within a certain margin away from the hyperplane. SVMs were first designed for classification problems, where the model learns a hyperplane that separates the different classes with the greatest margin. SVM for regression is a modification of the original SVM used for predicting a continuous output value (Forrester and Keane, 2009).

Unlike SVM for classification, which tries to separate the data with a clear margin, SVM for regression uses a technique known as epsilon-insensitive loss to allow some data points to be within the margin of error. The algorithm then tries to find the hyperplane to minimize the error between the predicted output values and the actual output values of the training set.

SVMs are uncommon as surrogate models in culture media optimization literature but have been used in other applications (Chintalapati et al., 2013). Despite limited usage in culture media optimization literature, it is generally held that SVMs are less prone to overfitting for smaller datasets compared to ANNs (Wilson, 2008) which could be an advantage in culture media optimization problems where there is a lower amount of data.

### 6.2 Acquisition functions

#### 6.2.1 Predicted value

Predicted value is the most straightforward acquisition function. It essentially treats the surrogate model as a faithful predictor of the actual objective function. Thus by optimizing the predicted value of the surrogate model, the hope is that the solution found is likely a near-optimum point (Forrester and Keane, 2009).

Predicted value is the most common acquisition function used for most surrogate models except for GPs. In culture media optimization, it has been used in combination with RSMs, ANNs and other models.

The drawback is that the surrogate model may give wrong predictions that deviate from the true objective function value by a large amount.

#### 6.2.2 Expected improvement

For surrogate models that can provide confidence estimation of prediction, alternative acquisition functions that incorporate prediction uncertainty can be used instead. This helps to address the problem of poor predicted values in less sampled regions.

The expected improvement (EI) acquisition function is designed to balance between exploration and exploitation of the search space (Forrester and Keane, 2009). Based on the predicted value of the surrogate model, EI favors points that have a high probability of improving the current best solution by considering the difference between the current best solution and the predicted value of the objective function at a new point. At the same time, EI also favors points that are uncertain, measured by the standard deviation of the predicted value of the objective function at a new point. Points that have a high standard deviation are more uncertain, and therefore more likely to provide new information about the search space. Thus, by considering both the potential improvement of a new point and the uncertainty of the prediction, EI acquisition function provides both exploitation and exploration.

EI is computed as such:

$$EI\left(x\right)=\left(f_{\min}-\hat{f}\left(x\right)\right)\Phi \left(\frac{f_{\min}-\hat{f}\left(x\right)}{s}\right)+s\phi \left(\frac{f_{\min}-\hat{f}\left(x\right)}{s}\right)$$

where $\Phi \left(.\right)$ represents the standard normal density function; $\phi \left(.\right)$ represents the probability distribution function; $\hat{f}$ is the surrogate model predictor; $f_{\min}$ is the current best functional value and s is the standard deviation (Bhosekar, 2020).

GPs provide an uncertainty estimate of its predictions inherently, and EI can be easily applied as the acquisition function and is often the choice by default. For typical ANNs used in regression, the uncertainty estimate is not available. However, one technique for uncertainty estimation is through measuring the variance in predictions between an ensemble of neural networks each trained separately on the same data. With this uncertainty estimate, EI can then be applied as the acquisition function (Lim et al., 2021). So far, in culture media optimization, EI as an acquisition function has only been used in conjunction with GPs. Given that ANNs are commonly used as the surrogate model in many culture media optimization studies, it may be useful to investigate using EI as the acquisition function on an ensemble of ANNs as the surrogate model.

#### 6.2.3 Custom functions

Some works have designed custom acquisition functions that incorporate other aspects relevant to their goals of media design. For example, Cosenza et al. (2022) used a custom acquisition function that also accounts for the cost of producing the media, converting a multi-objective optimization problem into a single objective problem.

### 6.3 Acquisition methods

#### 6.3.1 Analytical solution

For some surrogate models, namely, polynomial response surfaces, analytical solutions for the optima can be obtained by solving for the stationary points of the polynomial if they exist and checking the nature of the stationary points. This is known as canonical and ridge analysis (Dean et al., 2017).

One possible problem with this approach is that we may be interested in acquiring multiple points instead, which is more easily achieved through population-based metaheuristics. This may be why many papers chose to use methods such as genetic algorithms to optimize the predicted value of polynomial response surfaces rather than using an analytical solution.

#### 6.3.2 Local optimization methods

##### 6.3.2.1 Nelder-Mead Downhill simplex

Nelder-Mead downhill simplex is a direct search algorithm that optimizes by constructing a nondegenerate simplex in the search space and uses rules of evolving the simplex to drive the search (Lewis et al., 2000). A simplex refers to a set of *n*+1 points in an *n-*dimensional space.

In simplex search, the worst point of the simplex is reflected through the centroid of the opposite face of the simplex. If the new point improves upon the worst point, it is kept to form the new simplex. If not, then the next worst point of the simplex is used to reflect and generate a new point. If all new points do not improve compared to the existing simplex, the lengths of the edges adjacent to the current best vertex are reduced by half. This process can be thought of as a variation of the method of steepest descent where the direction of movement is the opposite direction to the gradient of a plane fitted to the simplex points.

Nelder-Mead downhill simplex can be used as a local optimization method to optimize the acquisition function, as used in Tripathi et al. (2012).

##### 6.3.2.2 BFGS and L-BFGS

BFGS (Broyden–Fletcher–Goldfarb–Shanno) is a widely used local optimization algorithm used for solving non-linear optimization problems. BFGS belongs to a family of Quasi-Newton methods, which are approximations of the Newton-Raphson method. The BFGS algorithm uses a limited number of gradient evaluations to approximate the Hessian matrix (describes the curvature of the function at a given point) and it updates this approximation at each iteration. L-BFGS (Limited-memory BFGS) is a variant of BFGS that is more suited for optimizing problems with many variables. BFGS stores a dense n×n approximation to the inverse Hessian (*n* is the number of variables), whereas L-BFGS stores a limited number of vectors for the approximation (Liu and Nocedal, 1989). Quasi-Newton methods such as BFGS are often the default choice in optimization libraries and are reliable in finding the local optima of smooth functions. It is therefore used as the default optimizer in many Bayesian optimization libraries such as GPyOpt, SMT, and scikit-optimize.

##### 6.3.2.3 Drawbacks of local optimization

Local optimization will only converge upon the local optima close to the initial point of search. When the surrogate model is highly non-linear and contains multiple local optima, it is difficult to find the global optimum of the acquisition function. This could be addressed by either running multiple instances of local optimization starting at random locations or using global optimization methods.

#### 6.3.3 Global optimization methods

Any of the optimization algorithms introduced in section 5 can optimize the acquisition function including genetic algorithm and differential evolution. Here we introduce two others that have been used in culture media optimization.

##### 6.3.3.1 Simulated annealing

Simulated annealing (SA) is a metaheuristic algorithm inspired by the annealing process of materials when cooled down from high temperatures. As the temperature of the material is slowly lowered, the atoms settle to a new configuration that has a lower internal energy. The initial starting position is thought of as a local minimum. The heating of the materials translates to replacing the current solution with new random solutions. The new solutions may be accepted based on a probability computed on the resulting function value decrease as well as a ‘temperature’ measure which is slowly decreasing as iterations increase. The temperature measure allows for solutions that have higher objective function values to be accepted which avoids trapping in local minima.

SA has been used to optimize the acquisition function when ANN or RSM is used as a surrogate model(Aquino et al., 2016; Parkhey et al., 2017; Dhagat and Jujjavarapu, 2021).

##### 6.3.3.2 DYCORS

DYCORS (Dynamic Coordinate Search using Response Surface Models) is an optimization algorithm used for solving non-linear and non-convex optimization problems. It is a variant of the coordinate search method and uses a dynamic selection of coordinates for optimization on radial basis response surface, which allows it to converge faster than traditional coordinate search methods.

The DYCORS algorithm starts with a feasible point and at each iteration, it selects a subset of coordinates to optimize based on the gradient information. Then, it performs a line search along the selected coordinates to minimize the objective function. The algorithm continues this process until a stopping criterion is met. The selection of coordinates is done dynamically, as the algorithm progresses, this allows it to adapt to the structure of the problem and avoid getting stuck in poor local optima. DYCORS has been used to optimize on a RBFNN (Cosenza et al., 2021).

#### 6.3.4 Strategies for acquiring multiple candidates

When proposing a new batch of candidates, having a diversity of candidates helps to explore the solution space better and avoid duplicate testing of similar candidates. If factors are continuous values, candidates can be technically different but have arbitrarily small differences. Hence when proposing candidates using the acquisition function, several strategies may be employed to promote diversity.

**FIGURE 4:**
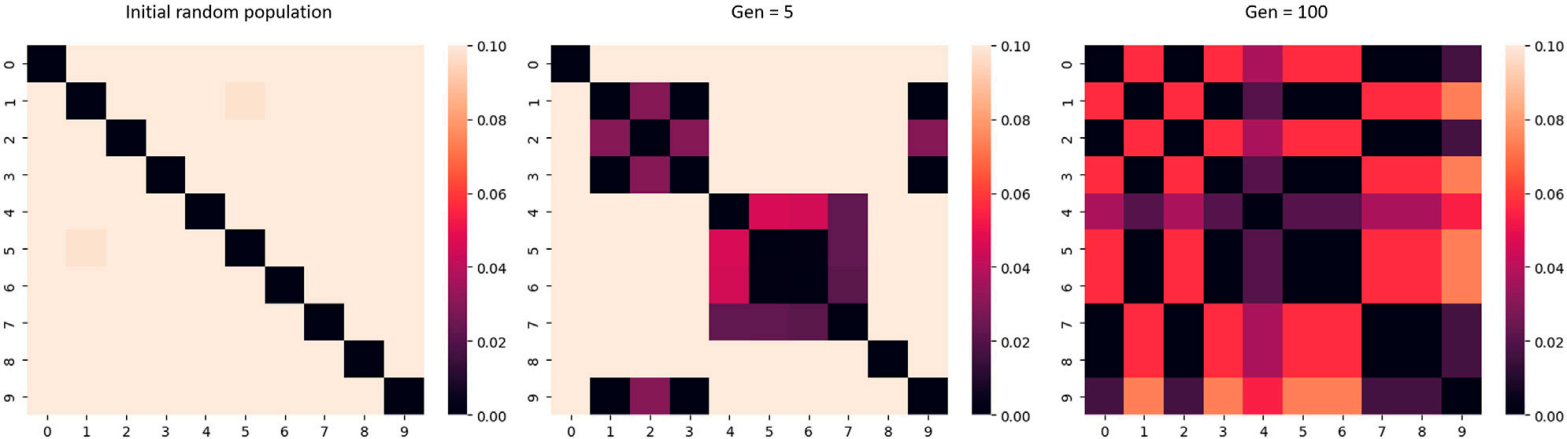
Heatmap of the L1 distance, where 0 represents the same value. As the number of generations of the algorithm increases, the diversity of the candidate population decreases.

Early stopping of the acquisition optimization algorithm can promote diversity for population-based metaheuristics, this is illustrated in Figure 4. When metaheuristics algorithms are allowed to run for many iterations, the solution population will generally converge upon an optimum. By stopping early, diversity in the solution population may be preserved. Truncated GA is one example of this strategy.

**FIGURE 5:**
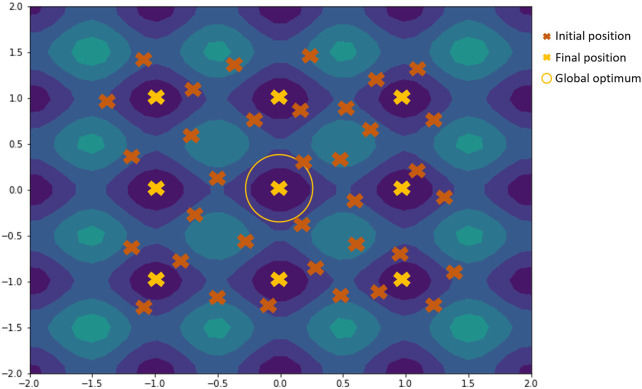
Illustration of how using multiple runs from random initial positions work on contour plot of the rastrigin function. Candidates from the initial positions would converge towards the nearest final position, with a few of them converging towards the global optimum.

Multiple runs of the acquisition optimization algorithm from random initial positions allow the acquisition of different local minima when using local optimization methods or non-population type metaheuristics like SA. As local optimization methods will generally converge upon an optimum that is closer to the initial position, by running this process multiple times but with different randomly selected starting positions, multiple local minima can be found if they exist in the function. An example is shown in Figure 5.

When EI is used as the acquisition function, another strategy of generating multiple candidates is the parallel efficient global optimization method with*qEI* criterion. The *q*-EI criterion was used to choose q ∈ N points, where q refers to the number of candidates and N refers to the population size. Instead of directly optimizing *q*-EI, Ginsbourger et al. (2008) proposed the use of two heuristics methods, Kriging Believer and Constant Liar as candidate designs. The *q*-EI criterion is subsequently used to choose between the two.

## 7 Characteristics of culture media optimization problems

There are several key characteristics of culture media optimization problems. These should be taken into consideration when choosing the method of optimization and is what differentiates this problem from others.

Firstly, the number of iterations for optimization is limited. This results from the fact that cell culture experiments are time-consuming and therefore experimental groups cannot afford to optimize for a large number of iterations. Many works perform a one-step optimization, where only an initial design of experiment was cultured, followed by one batch of proposed candidates based on the initial DOE. The average number of iterations performed across all works was 2.73 (including one-step optimization) and 6.22 (excluding one-step optimization). In most benchmarking of black-box optimization algorithms, the number of iterations far exceeds what can be afforded for culture media optimization.

Because of this, optimization algorithms that are slow in improvement or convergence are not suitable. Based on studies that compare the two, a surrogate model-based approach may be more suitable as the rate of improvement tends to be higher than metaheuristics algorithms in early iterations.

Secondly, the number of candidates is limited and dependent on the nature of the cell culture and available experiment equipment. For example, if one were to use the standard cell culture plate size of 96 wells with at least three replicates per candidate, that would limit the experiment to a maximum of 32 candidates, not accounting for limitations like the edge effect (Mansoury et al., 2021). Of course, researchers need not be restricted to using one plate and can also use other multiwell plates like 384-well or 1536-well plates, however, these come with their own limitations such as format restrictions of the equipment available. The average batch size used in all the works surveyed was 27.01.

Given the limitations in both iterations and batch size, the number of variables that can be optimized given certain experiment budget while achieving statistical power is an important consideration in media optimization experiments. In all works surveyed that used a surrogate model, the average number of candidates tested in total was found to be 40.46, with 7.40 candidates tested per factor. The average number of factors used was 8.10 (including one-step optimization) and 14.44 (excluding one-step optimization).

For experiments that use a 2nd order polynomial RSM for optimization, a typical number of factors used is 5. Using the average number of candidates tested, this gives roughly 2 datapoints per coefficient for fitting, which is sufficient to achieve power of approximately 0.8 with a R^2^ of 0.5 and a significance level of 0.05. However, with 6 factors, achieving the same power would need 50 candidates. It is important to consider the experimental budget and the need for sufficient statistical power when designing experiments to fit the response surface.

For non-parametric approaches or other machine learning models, validation sets can be used to estimate the accuracy of the model and determine if the number of datapoints is sufficient. Empirical rules of thumb may also be used as guides to decide on number of factors to choose given certain budget or conversely the number of experiments given certain number of factors. For example, a common guideline is to have data 10 times the number of factors. For metaheuristics algorithms, several studies (Bolufé-Röhler and Chen 2013; Chen et al., 2015) have looked at how population size affects performance at different dimensions. However, these have focused on metrics such as convergence time and closeness to optima after convergence, which is not entirely relevant to media optimization. Nevertheless, we may reference some guidelines such as having a population size larger than and preferably 10 times the dimension (Bolufé-Röhler and Chen 2013; Mallipeddi and Suganthan 2008).

Lastly, another key factor to consider is the effect of passaging. Often, bioprocesses require cells to be passaged continually for a few rounds. After each passage, the characteristics of the cells change which likely results in a change in the true function of the optimization problem. These characteristics include key gene functions, morphology, proliferation rate, and expression levels (Hughes et al., 2007). Currently, none of the black-box optimization algorithms account for this. One interesting approach used by Cosenza et al. (2022) was to include information about cell count by incorporating ‘low-fidelity’ information sources such as biochemical assays and ‘high-fidelity’ information sources like cell proliferation rate over one passage with a Bayesian optimization tool, thereby including single-passage and multiple-passage information into culture media optimization problems and accounting for the change in cell characteristics.

## 8 Trends in existing literature

The use of optimization algorithms for culture media optimization was first reported in 1992 by Freyer et al. (Dirk Weuster-Botz and Wandrey 1995), where GA was used to optimize media for formate dehydrogenase production in *Candida boidinii.* In the late 1990s and early 2000s, many researchers followed suit and started using GA for culture media optimization problems (Viennet et al., 1996; Patil et al., 2002; Marteijn et al., 2003; Bapat and Wangikar, 2004). Notably, Cockshott & Hartman (2001) were the first to implement PSO for the purpose of fermentation media optimization, where they hypothesized that PSO would perform better than GA based on previous reports that PSO performs better for smaller population sizes and converges to the optimum faster compared to GA. SBO for culture media optimization was first described by Coleman et al. (2003) where they used an ANN ensemble surrogate model to optimize *Escherichia coli* fermentation, based on previous studies in enology and other fermentation processes. Since then, many researchers have used optimization algorithms for the purpose of culture media optimization.

Of all the research articles available on the topic, approximately 70% reported using an SBO approach, of which roughly 43% used ANN as the surrogate model, PV as the acquisition function, and GA as the acquisition method (SBO(ANN)-PV-GA). The reason for the popularity of this method is unclear; many papers do not cite their reasons for choosing a particular model over others. Most of the papers compare SBO(ANN)-PV-GA to either classical methods such as OFAT screening (Desai et al., 2006) or to the use of statistical RSM as a surrogate (Desai et al., 2008; Pal et al., 2009; Baskar and Renganathan, 2010; Du et al., 2012; Gurunathan, 2012). Some cited the ability of ANN to excel in pattern recognition and modeling nonlinear relationships (Haider et al., 2008; Singh et al., 2008; Pal et al., 2009; Du et al., 2012; Peng et al., 2014) and its ability to work well with a small number of candidates (Desai et al., 2008) and noisy data (Pathak et al., 2015) as reasons why ANN models should be developed for biological systems. The next most popular approaches adopted were an SBO with RSM as the surrogate model, PV as the acquisition function, and GA as the acquisition method (SBO(RSM)-PV-GA); and direct optimization with GA. The papers that have compared SBO(ANN)-PV-GA and SBO(RSM)-PV-GA have usually concluded that ANN is a better model (Liu et al., 2009; Khaouane et al., 2012; Katla et al., 2019; Selvaraj et al., 2019; Suryawanshi et al., 2019) for media optimization problems, likely due to the nonlinear nature of the objective function.

**FIGURE 6:**
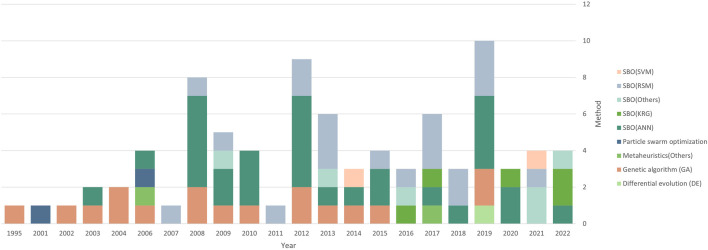
Trend in methods used over the years.

Some less commonly used methods include neuro-fuzzy networks as a surrogate model (Aquino et al., 2016), elastic net regularized general linear model as a surrogate model (Grzesik and Warth, 2021), and ensemble modeling (Liu and Gunawan, 2017). Interestingly, most researchers opted for parametric approaches as opposed to non-parametric approaches like GP or SVM, since nonparametric methods are known to outperform parametric methods for high-dimensional and nonlinear data (Liu et al., 2020). Figure 6 shows the trend in methods used over the years.

**FIGURE 7:**
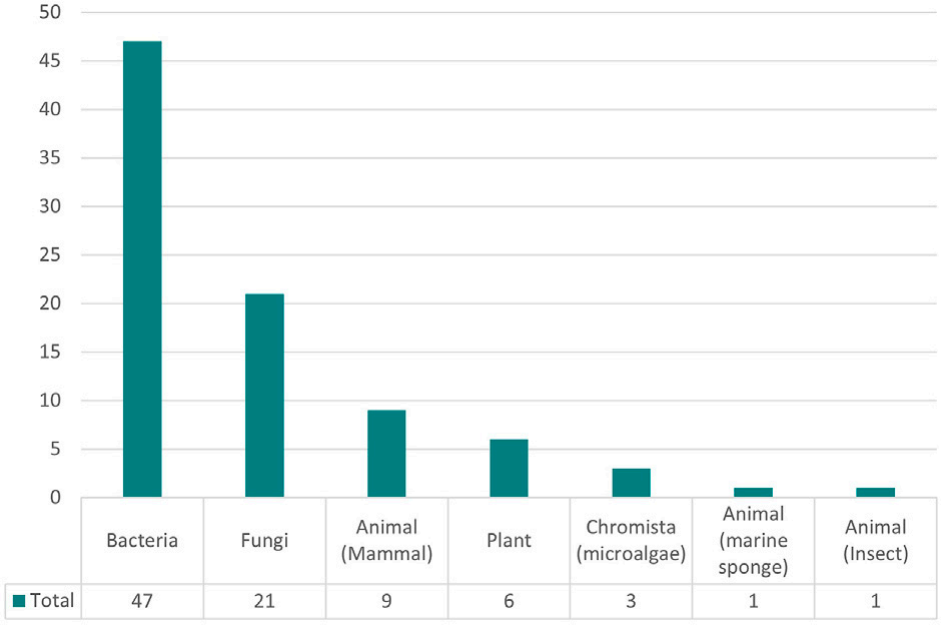
Distribution of types (kingdom) of organisms studied.

As shown in Figure 7, there is also an overrepresentation of bacteria and fungi as the cell line of choice in media optimization studies, due to the interest in optimizing the fermentation process in yeast strains. Despite rising interest in cultured meat production and the importance of antibody production, mammalian cells have not been studied extensively in media optimization research.

## 9 Common challenges

### 9.1 Noise in experiment results

It is inevitable that noise exists in biological experiment readouts. Having noisy experimental results is detrimental to optimization by hampering the ability and speed of the algorithm to converge. For example, in metaheuristics algorithms, the next-generation of candidates is typically generated from high-performing candidates. With noise, high-performing candidates may be spurious and perform poorly on average when tested repeatedly. This will lead to poor candidates generated for the next iteration, and reduce the rate of improvement. Similarly for surrogate models, noisy training data may result in a surrogate model that predicts the true function value (without noise) poorly.

Measures to reduce noise can be implemented in both the experimental and algorithmic design. Biological replicates can be used for each media formulation and the response values can be averaged across replicates, reducing variability in experimental results and providing the optimization algorithm with less noisy response values.

In terms of algorithm choice, many studies (Rakshit et al., 2017; Sudholt, 2021) have looked at how noise affects the performance of various methods. Some metaheuristics algorithms are more sensitive to noise, for example, Krink et al. (2004) found that standard DE performance degrades more than other metaheuristics on noisy functions.

For GPs, modeling with noise can incorporate the variability between replicates into the modeling process. The noise associated with the response may be either homoscedastic noise if noise variances are similar across observations, or heteroscedastic noise if noise variances vary across the observations. If the response measuring equipment is known to have a consistent range of uncertainty within the range of the response, then modeling with homoscedastic noise would suffice. Otherwise, estimates of the noise variance would be computed using the replicates of each candidate separately and heteroscedastic noise incorporated into the GP model.

### 9.2 Exploration and exploitation in proposing candidates

The trade-off between exploration and exploitation is a common challenge in optimization. Excessive exploration leads to slow convergence, while excessive exploitation increases the probability of being trapped in local optima. The design of most optimization algorithms implicitly tries to balance between these two needs. For example, in genetic algorithms, the selection and cross-over steps are designed to exploit by proposing new candidates that are similar to previous high-fitness candidates; the mutation step is designed for exploration by allowing for random changes in the candidates to increase diversity in the population. It would be helpful for practitioners to understand which hyperparameters of their algorithm of choice controls the exploration-exploitation tradeoff and adjust them accordingly.

For direct optimization methods, more emphasis on exploitation may be helpful given the limits of experimental capacity. A fast improvement on local optima to obtain “good enough” solutions could be sufficient for certain experiments.

When optimizing on the acquisition function of the surrogate model that occurs*in silico*, it may be advisable to allow more exploration to find the global optima of the acquisition function since the number of iterations is not limited. Note that this does not mean that the candidates should concentrate around the global optima of the acquisition function as this is in fact excessive *exploitation* behavior on optimizing the actual function. As discussed in Section 6.3.4, diversity should still be preserved as the surrogate model is unlikely to be accurate at the start and to avoid very similar candidates.

## 10 New directions in research

With the expansion of the cultured meat industry and the increasing need for therapeutic antibody discovery, media optimization in more mammalian cell contexts would be beneficial. Optimization for mammalian cell culture media poses a more complex challenge as compared to microbial culture media due to higher number of components. This means the existence of more and potentially higher-order interactions between components, leading to a much more complex objective function. Increasing the number of interactions would also complicate the screening process - the methods currently implemented may not effectively identify the most important components when the number and complexity of interactions between components in the media increase.

In most studies, researchers have used a single measure of enzyme of interest activity or a measurement that is representative of the protein expression level like fluorescence from GFP expression. However, these measurements may not always accurately indicate the actual protein yield in the cells. A related problem is the use of lower-fidelity measurements to reduce experimental burden while complementing with fewer high-fidelity but expensive or time-consuming measurements. In such cases, the need to synthesize multiple information sources related to the outcome will be useful. Cosenza et al. (2022) introduced the use of multi-information source Bayesian optimization where information from multiple assays was combined to measure cell growth. This is achieved with a modification to the kernel of the GP, by adding an additional Gaussian kernel to account for the deviation of the lower-fidelity measurement away from the true function value (assumed to be equal to high-fidelity measurement). The allocation of each batch of candidates between high and low fidelity for measurement is decided through the combination that produces the highest multi-point expected improvement.

Another possible addition to media optimization problems would be multi-objective optimizations. Often, optimization problems involve multiple objectives that sometimes conflict, such as maximizing cell growth and minimizing cost, that can be optimized simultaneously. Examples include maximizing metabolic activity (Havel et al., 2006) and maximizing cell count (Kim and Audet, 2019). Multi-objective optimization would allow for the prediction of a more universal solution set that can be chosen from depending on the unique constraints or subjective desired outcomes. For more information on multi-objective optimization, we refer interested readers to (Collette and Siarry 2004).

Lastly, there is also a lack of use of newly developed algorithms in culture media optimization research. Recently, Yoshida et al. (2022) and Tachibana et al. (2021) have used deep neural networks (DNN) with 4 hidden layers for fermentation media optimization and have found that they perform better than other machine learning models. Although deep learning has gained recent popularity in many other applications, it remains to be seen if DNNs will improve outcomes in media optimization application considering limited studies. There are other advanced optimization methods worth implementing for media optimization, including deep kernel regression as surrogate models (Wilson et al., 2015), and derivative-free reinforcement learning methods like neuroevolution of augmenting topologies (NEAT) (Qian and Yu, 2021). In this review, we will not be exhaustively expanding upon or analyzing the methods not currently found in culture media optimization literature.

## 11 Comparison of methods with simulation experiment

As described in Section 2.3, simulation experiments were done to compare the available methods for media optimization problems. To compare the algorithms’ performance across all functions, an average performance score is defined as follows:

$$Normalised\;functional\;value=\frac{y_{initial}-y_{final}}{y_{initial}-Global\;optimum}$$

where y_initial_ refers to the functional value at the first iteration; y_final_ refers to the functional value at the last iteration and the global optimum is the near-optimal solution found by running DE for 1000 iterations. The average performance score for an algorithm across all different functions can be defined as the mean of the scores on individual functions.

The following methods were compared throughout our simulation experiments:

Metaheuristics methods.

• Genetic Algorithm (GA)
• Differential Evolution (DEbest1bin)
• Particle Swarm Optimization (PSO)

Various SBOs with differing surrogate models, with PV as the acquisition function and GA as the acquisition method.

• Second-order polynomial (SBO(2OP)-GA-PV)
• Kriging (SBO(KRG)-GA-PV)
• Multi-Layer Perceptron (SBO(MLP)-GA-PV)
• Support Vector Machine (SBO(SVR)-GA-PV)

Kriging SBO with a different acquisition function.

• EI (SBO(KRG)-GA-EI)

Kriging SBO with different acquisition methods, with PV as the acquisition function.

• Truncated GA (maximum number of generations = 10) (SBO(KRG)-truncGA-PV)
• Truncated DE (maximum number of generations = 10) (SBO(KRG)-truncDE-PV)
• L-BFGS (SBO(KRG)-L-BFGS-B-PV)

These models were chosen as they were the most popular methods used in the field of culture media optimization. Kriging was chosen as the model for comparison against other infill strategies as according to our preliminary data, it was the best performing surrogate model. As explained in Section 2.3, the experiments were conducted at three levels of dimensions to represent the varying complexities of different types of culture media. The performance of the methods was also compared in experiments with noise to evaluate its effect on various methods.

For the low dimension experiment (dim = 5), a comparison of three different DOE methods, LHS, CCD and BBD was also done to understand how each DOE affects the performance of the methods. BBD was supplemented with LHS to ensure an equal number of candidates across the DOE. This comparison was only done for the low dimension experiment as the number of candidates would be too high and unfeasible to replicate experimentally for higher dimensions. BBD was found to be the best performing DOE (Supplementary Table S2).

**FIGURE 8:**
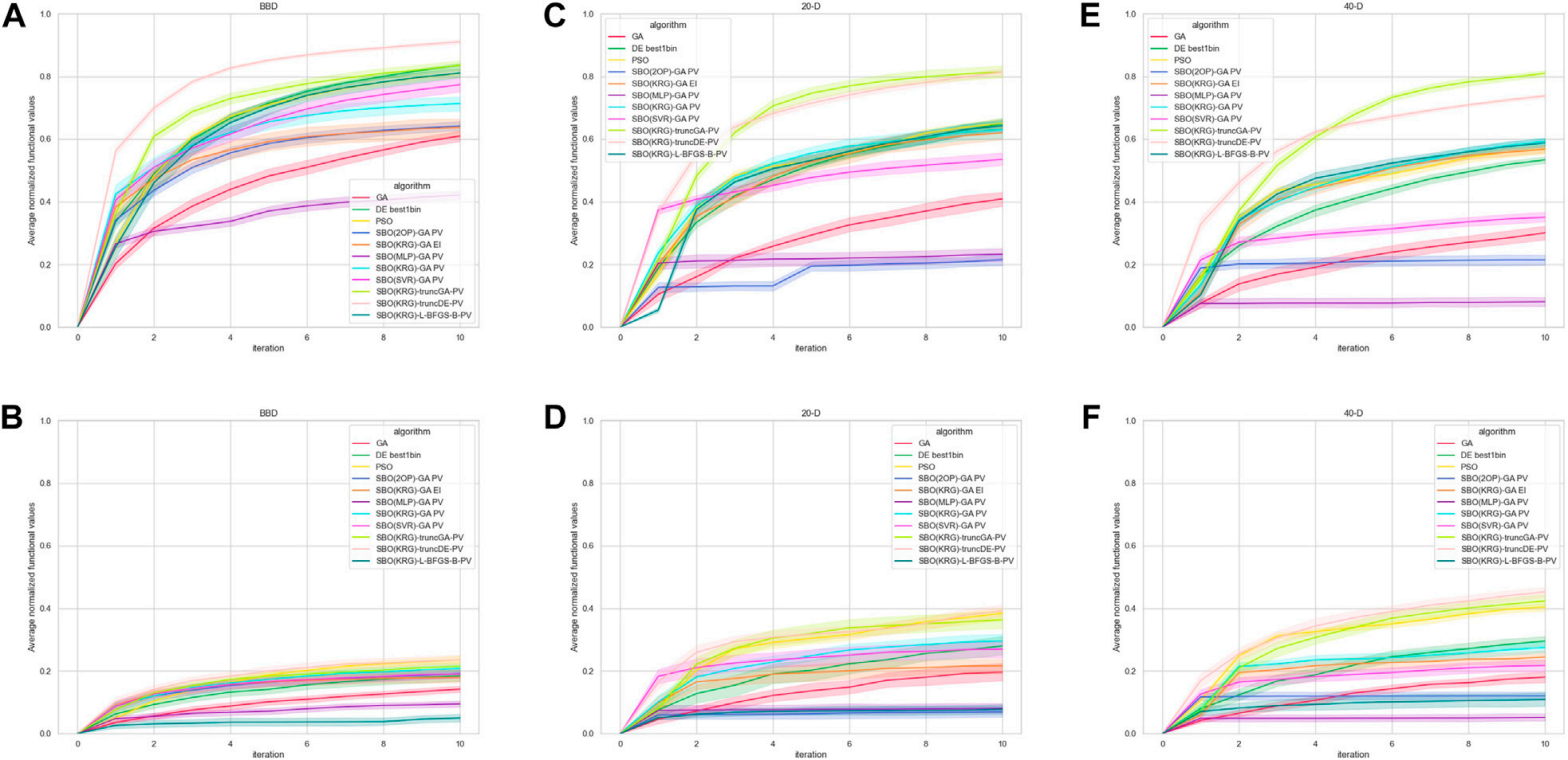
Performance of the methods in each context, represented by average normalized functional value over each iteration. Bands represent the 95% confidence interval. Unless otherwise stated, the DOE used is LHS. **(A)** Low dimension experiment results, with BBD as the DOE and without additive noise **(B)** Low dimension experiment results, with BBD as the DOE and additive noise **(C)** Medium dimension experiment results without additive noise **(D)** Medium dimension experiment results with additive noise **(E)** High dimension experiment results without additive noise **(F)** High dimension experiment results with additive noise.

Figure 8 shows the performance of the various methods in each respective context. In the ‘noiseless’ experiments, some variation exists in output values across the replicates due to the stochastic nature of the methods. The relative performance of the methods does not differ significantly across the different DOE in the low dimension experiments (data not shown). According to Figure 8A, in the low dimension experiments without additive noise Kriging with truncated DE as the acquisition method and PV as the acquisition function is the best method across all iterations.

In the experiments with added noise, the performance of all the methods deteriorates significantly. However, SBO(KRG)-truncDE-PV remains the best performing method across all iterations (Figure 8B), with PSO being a close second at higher iterations. According to Figure 8B, methods such as SBO(2OP)-GA-PV and SBO(SVR)-GA-PV perform comparatively well until iteration 4, which is congruent with the many papers that had success with RSM methods with just one iteration (Liu et al., 2009; Khaouane et al., 2012; Parkhey et al., 2017). Another popular method, SBO(MLP)-GA-PV, however, performed less well compared to the others. The SBO with the acquisition method as a local optimizer (SBO(KRG)-L-BFGS-B-PV) performed the worst consistently across all iterations and its performance seems to degrade significantly in the presence of noise (Figures 8A, B).

Figure 8C shows the results for the medium dimension (dim = 20) experiments without additive noise. Interestingly, in this case SBO(KRG)-truncGA-PV performs better than SBO(KRG)-truncDE-PV at certain iterations. These two methods perform significantly better than the other methods, while the performance of PSO, DE, SBO(KRG)-L-BFGS-B, SBO(KRG)-GA-PV and SBO(KRG)-GA-EI is comparable across iterations.

Similar to the low dimension experiments, the performance of all the methods deteriorates with the addition of noise. The performance of SBO(KRG)-truncGA-PV, SBO(KRG)-truncDE-PV and PSO are comparable from iteration 4 onwards, with significantly better performance as compared to the other methods. Notably, the performance of SBO(2OP)-GA-PV is much lower compared to its performance in the low dimension experiment.

The results of the high dimension (dim = 40) experiments without additive noise (Figure 8E) are very similar to that of Figure 8C, barring a significant deterioration in the performance of SBO(SVR)-GA-PV.

In the high dimension experiments with additive noise, SBO(KRG)-truncDE-PV is clearly the best choice over almost all iterations, with PSO as a feasible substitute.

Overall, the SBO methods with truncated acquisition methods seemed to triumph across all the contexts. This was likely due to the conserved diversity in the proposed candidates which prevented convergence to a local optimum, thus allowing the method to find a near-optimal solution faster. PSO has also fared relatively well across the experiments even with additive noise. Notably, the hypothesis that SVM would perform well with small datasets has generally held true. It is also surprising that SBO(KRG)-GA-EI did not perform better than its PV counterpart across all experiments, as EI helps to balance exploration of the input space with exploitation. This could be because the parameters set for GA allow it to strike a good balance between exploration and exploitation such that the use of EI as an acquisition function disrupted this balance, resulting in too much exploration and thus wasting resources on solutions that are less likely to be near-optimal. ANN did not seem to perform well across the experiments. The popularity of ANNs and their improved performance against RSM as reported in many media optimization studies is therefore surprising. This could be because most such studies conduct a one-step optimization which is not sufficient to determine the performance at higher number of iterations. Another reason could be the lack of hyperparameter tuning in the implementation of the MLP or other differences in implementation.

Table 1 shows a compilation of all the methods used in culture media optimization literature and their corresponding rankings according to the normalized values reached at the 10th iteration; and the number of iterations needed to achieve the 1st iteration normalized value reached by SBO(KRG)-truncDE-PV, averaged across all noisy experiments. There is a lack of papers that have used the best performing methods we have found, meaning that the use of these methods could result in better improvements than reported.

**TABLE 1:** Compilation of all the methods used in culture media optimization. Rankings are listed for methods that were evaluated in this paper, based on final functional values across experiments with additive noise, ranked 1–10 from best to worst. Iterations to reach set value is the number of iterations each method took to reach to achieve the normalized value achieved by the best performing method, SBO(KRG)-truncDE-PV in the first iteration.

Type	Surrogate Model/Metaheuristic	Acquisition method	References	Count		Ranking		Iterations to reach set value
Metaheuristic	GA	-	Garlapati et al., 2010; Patil et al., 2002; Marteijn et al., 2003; Bapat and Wangikar, 2004; D. Weuster-Botz and Wandrey, 1995; Hofer et al., 2004; Hutwimmer et al., 2008a (2008b),Sarma et al., 2009; Kucharzyk et al., 2012; Tišma et al., 2012; Chauhan et al., 2013; Singha and Panda, 2014; Camacho-Rodríguez et al., 2015; Munroe et al., 2019; Brinc and Belič, 2019; Heylen et al., 2006	16		8		7
Metaheuristic	PSO	-	Cockshott and Hartman, 2001; Huang et al., 2007; Garlapati et al., 2010	3		3		2
Metaheuristic	Multi-objective GA	-	(Havel et al., 2006)	1		-		
Metaheuristic	SA	-	(Dhagat and Jujjavarapu, 2021)	1		-		
Metaheuristic	DE	-	(Kim and Audet, 2019)	1		5		3
SBO	GP	Local optimizer	(Cosenza et al., 2022)	1		10		>10
SBO	ANN	GA	(Desai et al., 2006; Desai et al., 2008; Pal et al., 2009; Baskar and Renganathan, 2010; Du et al., 2012; Haider et al., 2008; Singh et al., 2008; Peng et al., 2014; Pathak et al., 2015; Suryawanshi et al., 2019; Selvaraj et al., 2019; He et al., 2008; Subba Rao et al., 2008; Singh et al., 2009; Guo et al., 2010; Gurunathan and Sahadevan, 2011; Kana et al., 2012; Baskar and Renganathan, 2010; Kana et al., 2012; Rekha et al., 2013; Zhou et al., 2015; Wei et al., 2017; Kumar et al., 2017; Pandey et al., 2018; Katla et al., 2019; Joji et al., 2019; Prabhu et al., 2020; Zhang et al., 2020; Imandi et al. n.d.)	29		9		>10
SBO	ANN	PSO	(Khaouane et al., 2012)	1		-		
SBO	ANN	hybrid GA	(Coleman et al., 2003)	1		-		
SBO	ANN	Local optimizer	(Tripathi et al., 2012; Dhagat and Jujjavarapu, 2021)	2		-		
SBO	RSM	GA	(Parkhey et al., 2017; Selvaraj et al., 2019; Kumar et al., 2017; Maiti et al., 2011; Moorthy and Baskar, 2013; Y; Singh and Srivastava, 2013; Kanimozhi et al., 2017; Shirodkar and Muraleedharan, 2017; Srivastava et al., 2018)	9		7		>10
SBO	RSM	DE	(Eswari et al., 2013)	1		-		
SBO	RSM	PSO	(Khaouane et al., 2012)	1		-		
SBO	RSM	SA	(Parkhey et al., 2017)	1		-		
SBO	RSM	Analytical Solution	(Abdel-Fattah et al., 2007; Abdel-Fattah, 2009; Burrows et al., 2009; Abbasi et al., 2013; Farag et al., 2018; Katla et al., 2019)	6		-		
SBO	RSM	Q2	(Burrows et al., 2009)	1		-		
SBO	RSM	Multi-objective GA	(Kumar et al., 2015; Unuofin et al., 2019)	2		-		
SBO	SVM	GA	(Xu et al., 2014; Hesami and Jones, 2021)	2		6		2
SBO	Random Forests	Exhaustive grid search, kmeans	(Grzesik and Warth, 2021)	1		-		
SBO	RBFNN	GA	(Tian et al., 2013)	1		-		
SBO	RBFNN	PSO	(Liu et al., 2009)	1		-		
SBO	RBFNN	truncated GA	(Zhang and Block, 2009)	1		-		
SBO	RBFNN	truncated GA + DYCORS	(Cosenza et al., 2021)	1		-		
SBO	Neuro-fuzzy networks	SA	(Aquino et al., 2016)	1		-		
SBO	Bayesian-regularized NN	Batch relative information gain	(Zhang and Block, 2009)	1		-		
SBO	Bayesian-regularized NN	PSO	(Khaouane et al., 2012)	1		-		
SBO	Elastic net regularized general linear models	Exhaustive grid search, kmeans	(Grzesik and Warth, 2021)	1		-		
SBO	Non-parametric Regression with Gaussian Kernel	ranking pseudo-r square	(Zou et al., 2020)	1		-		
SBO	GRNN	Fruit fly optimization	(Salehi et al., 2021)	1		-		
SBO	GRNN	GA	(Jafari et al., 2022)	1		-		
SBO	Adaptive neuro-fuzzy inference system	GA	(Farhadi et al., 2020)	1		-		
SBO	GP	Markov Chain Monte Carlo	(Morschett et al., 2017)	1		-		
SBO	GP	Batch Contextual Local Penalization	(Kanda et al., 2022)	1		-		
SBO	GP	Full factorial in new expanded region	(Freier et al., 2016)	1		-		
SBO	GP	GA		0		4		2
SBO	GP	Truncated GA		0		2		2
SBO	GP	Truncated DE		0		1		1

Based on the results of the simulation experiments, we recommend Kriging SBO together with a truncated metaheuristics acquisition method as a highly competitive optimization method that would likely meet the needs of most culture media optimization experiments.

While the simulation experiments conducted were quite extensive, they were not exhaustive and were focused more on the methods proven to yield promising results. For example, methods such as variable neighborhood search, simulated annealing and a variety of direct search methods like mesh adaptive direct search have not been evaluated in the culture media optimization context. Given that PSO performs well as a metaheuristic, it would also be interesting to know whether using it as an acquisition method would improve the current best method of SBO(KRG)-PV, or in general for other surrogate models. It would also be useful to verify if the truncated acquisition methods improve the performance of surrogate models other than Kriging. Furthermore, the number of generations for truncated acquisition methods could be optimized such that the diversity of the suggested candidates is maintained while also converging faster to the global optimum. Lastly, while the BBOB test suite makes for a suitable alternative to time-consuming experimental validation, the validity of these results should be proven through experimental validation.

## 12 Conclusion and outlook

Since its inception, cell culture is playing an increasingly important role in the production of various useful products including biopharmaceuticals, enzymes and chemicals. The market size for products manufactured with microbial fermentation is estimated to be USD 28.3 billion (Custom Market Insights, 2022) and the market size for biopharmaceuticals manufactured using animal cell systems is estimated to be USD 24.6 billion (‘Cell Culture Market Size, Share & Trends Report, 2022–2030’ 2018). In recent years, new applications are emerging in cell culture for food production known as cellular agriculture, which includes cultured meat, single-cell proteins and precision fermentation. The market for cellular agriculture is projected to grow to USD 515.2 billion by 2030 (Research 2021). In scalable cell culture production systems, culture media is often the crucial determinator of techno-economic viability through its effect on product yield and its role as the main contributor to production costs. For example, in monoclonal antibody production using animal cell cultures, culture media is estimated to account for 30%–40% of the production cost (Batista and Fernandes, 2015). Thus, optimizing the culture media for yield and cost is very important for the success of scalable commercial applications and for reducing the cost for many products.

Given the expansive literature on culture media optimization, we recognize the challenge researchers face in perusing existing works and selecting a suitable optimization methodology for their own cell culture application. In the absence of studies that summarize and compare between various available methods, it is common to default to methods that were used in similar studies when a better method may exist.

In this review article, we hoped to (i) provide a generalized framework for understanding and designing culture media optimization experiments; (ii) summarize and classify the large number of existing works; (iii) examine common challenges and algorithmic features that were designed and chosen during past efforts to such challenges; (iv) provide recommendations on the type of algorithm to use based on benchmark comparisons.

For standard applications of culture media optimization, the benchmark comparison may serve as a reference to help select the algorithm that is likely to perform well on generic unknown functions within experimental resource constraints. For applications where existing methods provide limited results, and are seeking to develop improved algorithms, we identify a few possible areas where efforts might be fruitful.

Development of better surrogate models is an area where advances in data-driven predictive models can be applied. As discussed in Section 10, advances in machine learning algorithms and deep learning may find useful application especially for higher dimension problems. From the benchmark experiments, the method of acquisition has a significant effect on optimization performance. Hence designing of better acquisition methods is also likely to yield improvements. Acquisition methods that can successfully balance exploitation and exploration through clever designs can achieve fast improvement while avoiding premature convergence. Other areas where new developments have been made are discussed in detail in Section 10.

In this review, we have focused exclusively on methods used in cell culture media that are knowledge-blind. However, it should be recognized that there are methodological limitations of relying on such optimization approaches where only media component concentrations/levels and output responses are collected and used. Biological data of cell response, such as the cell transcriptomes, or analysis of spent media can be used to enhance predictive surrogate models. Chemical information about the media components may also be incorporated into such models, opening up the possibility of building surrogate models that can generalize to other media components outside the original list of components used in the experiment. Various AI, bioinformatic and chemoinformatic approaches may find potential use in the discovery, design and optimization of cell culture media.
