# Supplementary material for: A review of algorithmic approaches for cell culture media optimization
Source: Front Bioeng Biotechnol. 2023 May 11;11:1195294. doi: 10.3389/fbioe.2023.1195294 (PMC10213948; doi:10.3389/fbioe.2023.1195294)
Supplement: Supplementary file 1 [file Table1.DOCX]

Supplementary Material

A review of algorithmic approaches for cell culture media optimization

Tianxun Zhou, Rinta Reji, Ryanjit Singh Kairon, Keng Hwee Chiam*

*** Correspondence:** Keng Hwee Chiam: [chiamkh@bii.a-star.edu.sg](mailto:chiamkh@bii.a-star.edu.sg)

# Supplementary Table 1: BBOB test suite functions and their properties

| No. | Function | Characteristics |
| --- | --- | --- |
| Group 1: Separable | | |
| 1 | Sphere | Unimodal, separable, symmetric |
| 2 | Ellipsoidal | Unimodal, separable, high conditioning |
| 3 | Rastrigin | Multimodal, separable, low conditioning, regular structure |
| 4 | Buche-Rastrigin | Multimodal, separable, regular structure , asymmetric |
| 5 | Linear Slope | Unimodal, separable |
| Group 2: Functions with low or moderate conditioning | | |
| 6 | Attractive Sector | Unimodal, low or moderate conditioning, asymmetric |
| 7 | Step Ellipsoidal | Unimodal, low/moderate conditioning, many plateaus |
| 8 | Rosenbrock | Unimodal or bimodal, low or moderate conditioning |
| 9 | Rosenbrock, rotated | Unimodal or bimodal, low or moderate conditioning |
| Group 3: Functions with high conditioning and unimodal | | |
| 10 | Ellipsoidal | Unimodal, non-separable, high conditioning |
| 11 | Discus | Unimodal, high conditioning |
| 12 | Bent Cigar | Unimodal, high conditioning |
| 13 | Sharp Ridge | Unimodal, high conditioning |
| 14 | Different Powers | Unimodal, high conditioning |
| Group 4: Multi-modal functions with adequate global structure | | |
| 15 | Rastrigin | Multimodal, non-separable, low conditioning, regular structure |
| 16 | Weierstrass | Multimodal, repetitive, adequate global structure |
| 17 | Schaffers F7 | Multimodal, low conditioning, asymmetric |
| 18 | Schaffers F7 | Multimodal, moderate or high conditioning, asymmetric |
| 19 | Griwank-Rosenbrock | Multimodal, low or moderate conditioning |
| Group 5: Multi-modal functions with weak global structure | | |
| 20 | Schwefel | Multimodal, weak global structure |
| 21 | Gallagher G.101-me Peaks | Multimodal, low conditioning, weak global structure |
| 22 | Gallagher G. 21-hi Peaks | Multimodal, moderate conditioning, weak global structure |
| 23 | Katsuura | Multimodal, repetitive, weak global structure |
| 24 | Lunacek bi-Rastrigin | Multimodal, weak global structure |

Supplementary Table 1 shows the 24 BBOB test suite functions divided into 5 groups.

Supplementary Table 2: Average normalized final functional values across all methods for low dimension results

| DOE | Noise | Average Normalized Values across all methods |
| --- | --- | --- |
| LHS | No | 0.68 |
| BBD | No | 0.7 |
| CCD | No | 0.69 |
| LHS | Yes | 0.18 |
| BBD | Yes | 0.19 |
| CCD | Yes | 0.15 |

Supplementary Table 2 shows the performance of each DOE for the noiseless low dimension results. In the noiseless experiments, statistical DOEs fare better than LHS. BBD seems to perform slightly better than CCD, which performs slightly better than LHS. The overall performance has decreased in the experiments with noise, with BBD remaining the best performing DOE, whereas LHS seems to perform better than CCD in this context. For the purpose of culture media optimization with a low number of factors, BBD would be the recommended DOE.
